# Black Soldier Fly (*Hermetia illucens*) Microbiome and Microbe Interactions: A Scoping Review

**DOI:** 10.3390/ani14223183

**Published:** 2024-11-06

**Authors:** Shu-Wei Lin, Matan Shelomi

**Affiliations:** Department of Entomology, National Taiwan University, No 1 Sec 4 Roosevelt Rd, Taipei 106319, Taiwan

**Keywords:** bacteria, BSF, edible insects, insect farming, probiotics

## Abstract

Black soldier fly (*Hermetia illucens*, BSF) is farmed worldwide to convert organic waste into usable biomaterials. Studies on the larval microbiome have been carried out to check for symbiotic or pathogenic microbes and their respective functions and fates. Some studies tested these microbes for industrial applications, while others tested the effects of exogenous microbes as probiotics or for substrate pre-processing to improve larval fitness, bioconversion rates, or nutritional qualities. This review examined all peer-reviewed literature on these topics to consolidate many disparate findings together. It followed the PRISMA guidelines for scoping reviews. The results found no evidence of globally conserved core microbes, as diet strongly correlated with gut microbiome, but some genera appeared most frequently in BSF larval guts worldwide regardless of diet. The gut microbes undoubtably assist in digestion, including pathogen suppression, and so microbial probiotics show promise for future investigations. However, the common gut microbes have not been explored as probiotics themselves, which would be a promising direction for future work. The impacts of BSF bioconversion on pathogens varied, so each rearing facility should investigate and manage their pathogen risks independently. The data summarized in this study provide useful reference points for future investigations into BSF–microbe interactions.

## Simple Summary

The black soldier fly is rapidly becoming the most extensively farmed insect on the planet. The number of relevant publications is also growing exponentially. This review covers all the most recent, available publications on the subject of how this insect interacts with microbes. It consolidates the information on the insect’s gut microbiome and identifies species that could be used as probiotics for fly farmers. The review affirms past results that black soldier fly larvae do rely on gut microbes for digestion, but do not have a globally conserved core microbiome. Bacterial probiotics for the larvae show promise, but the most common gut microbe genera have so far not been tested as probiotics. This is a promising direction for future investigations.

## 1. Introduction

*Hermetia illucens*, commonly known as black soldier fly (BSF), is gaining increasing attention because its larvae (BSFL) can convert organic waste into valuable substances such as biodiesel or insect meal for commercial feed [1]. Some of this ability is thanks to the larvae’s own digestive enzymes, while some come from their gut microbes [2]. Transcriptomics data show BSFL expresses the same genes regardless of diet [3], so any enzyme necessary for a particular substrate that the flies lack would need to be produced by substrate or gut microbes or not at all. The microbiomes of larval guts and frass differ significantly from those of their diet [4,5], and researchers have tried to determine whether a core microbiome for BSFL exists [6,7,8]. These BSFL gut microbes themselves are also seen as a valuable product of BSFL production that could be isolated and used for other industries like animal feed supplementation [9,10], soil remediation or amendment [11,12], and bioenergy production [13,14,15,16,17].

Studying the BSFL microbiome has other purposes. As the larvae are an animal feed ingredient, it is crucial to know whether they could be contaminated during the waste conversion process in order to meet strict food safety standards [18]. While the larvae can suppress pathogen growth in the substrate even after they are removed from it [19], they can also carry pathogens and cause cross-contamination [20].

Another reason to study BSFL-associated microbes is that the gut or substrate microbiota affects larval development and bioconversion parameters [21,22]. Schreven et al. found that autoclaving the substrate reduced larval performance, and re-inoculating the substrate with its initial microbes restored normal performance [23]. Auger et al. found that the transcriptional profile of larvae changed extensively in a microbe-free environment [24]. Some of these changes are desirable from waste management or larval biomass production perspectives. For example, Suo et al. found that non-sterile larvae degrade aflatoxin B1 better than germ-free larvae [25]. Deliberate manipulation of the gut microbiome by introducing different species of bacteria into the substrate could thus produce desirable effects or minimize risks [26].

For these reasons, a large body of literature exists about BSFL–microbe interactions. However, some research siloing is present, with different research groups focusing on specific aspects of BSFL–microbe interactions (pathogens, probiotics, descriptive studies, mechanistic studies, etc.). To break down these silos [27] and promote the innovation highly desired in the BSF industry, this review summarizes the past studies on the BSFL gut microbiome regardless of sub-discipline. The goal is to provide a complete dataset of what has been discovered so far, covering all aspects of BSF–microbe interactions: core microbe(s), exploitable microbes, hygiene-concerned pathogens and substrate-borne detrimental elements, and BSFL probiotics and beneficial materials. While older reviews of individual aspects of the subject exist [28,29], and some reviews touch on these aspects but do not focus on microbes [30,31], this review differs in its comprehensiveness. Studies were not excluded on the basis of their methodology as in prior reviews. The hope was to produce a database from which disparate sources of information could be pulled to synthesize new knowledge: for example, determining from the gut microbiome studies what species would be best to target for a probiotics study.

Equally important as this paper’s novel comprehensiveness is the fact that this is the first review on BSFL–microbe interactions to follow the formal guidelines set out in the Preferred Reporting Items for Systematic Reviews and Meta-Analyses (PRISMA) Statement [32], which exist to improve transparency and scientific merit in reviews. Journals increasingly require that review papers follow the PRISMA guidelines [33]. This review also differs in that it explicitly included studies published in languages other than English, including several studies published in Chinese. This inclusivity is recognized as a new best practice for review papers [34]. Here, it addresses a linguistic gap in prior BSFL reviews and acknowledges the extensive research in BSF coming out of Chinese-speaking nations in the past two decades [35]. Finally, given the speed at which new discoveries are made in the field of BSFL, papers only a few years old can rapidly become out of date by no fault of their own [36], necessitating regular updates.

To summarize, the purpose of this work was to collate all studies covering any aspect of BSFL–microbe interactions, chart the extent of this research, identify key conclusions and research gaps, and finally put all the data into a convenient, single resource that future researchers can use.

## 2. Materials and Methods

### 2.1. Identifying and Selecting Relevant Studies

A scoping review was chosen as the method of inquiry, as the goal was to map the extent of disparate forms of literature and identify gaps [37,38]. Searches were made in April 2024 of the PubMed, Scopus, Web of Science, and CABI databases using the following search string: (“black soldier fly” OR “Hermetia illucens”) AND larvae AND (microb* OR bacteria* OR fung* OR microorganism*). These terms were chosen to include studies on eukaryotic microbes and viruses as well as bacteria. No limits were placed on the year of publication.

The results were uploaded to the Covidence platform, https://www.covidence.org/ (accessed on 29 October 2024) for systematic review management, including duplicate removal. Articles were eligible for inclusion if they investigated an aspect of BSFL–microbe interactions. These included studies on the natural gut microbiome of the BSFL, studies attempting to influence BSFL bioconversion through experimental addition of microbes, studies observing the effects of BSFL bioconversion on microbes in the substrate, and studies utilizing microbes isolated from the BSFL for other purposes. Conference proceedings, posters, review papers, and papers in predatory journals [39] were excluded. Papers for which only abstracts were available were excluded. To maximize diversity and inclusivity and correct linguistic biases in previous BSFL review papers, the search was not limited to English-language papers [34]. Included studies could be written in any of the languages the authors are collectively able to read: English, Chinese, French, and German. Note that all articles had English abstracts, and the search string used was in English. 

The titles and abstracts of articles were independently reviewed by two authors (S.-W.L. and M.S.). If articles were not excluded, then the articles went through a full-text independent review by two authors (S.-W.L. and M.S.). If disagreements arose, a third-party reviewer was consulted. Any relevant publications fitting the inclusion criteria identified from the full-text reviews of these articles that were not identified by the initial database searches were also included. No specific time frame was chosen for selection or data extraction processes, though all were completed in five months.

This paper meets the requirements of the PRISMA Extension for Scoping Reviews (PRISMA-ScR) checklist [40] and the protocol has been publicly registered at https://osf.io/5jm7b/ (accessed 6 November 2024).

### 2.2. Data Extraction

The data were analyzed via narrative synthesis [41]. The papers were sorted by type of study (BSFL microbiome surveys, experiments on the effects of beneficial microbes on BSFL, and experiments on BSFL’s effects on deleterious microbes). Relevant data were extracted by the lead author (S.-W.L.), reviewed by a second author (M.S.), synthesized in summary format, and charted in Microsoft Excel v16.66.1. For studies that involved identifying gut microbiome composition, the information charted for each experiment or sample type in each reference included the microbial taxa with more than 5% mean relative abundance at the end of each experiment, the substrate or diet, duration of the experiment, location of the study, method of microbiome detection (e.g., 16S sequencing, metagenomics), and any special manipulations involved (e.g., addition of antibiotics or salts). For experimental manipulation of microbiome studies, the information charted for each experiment or sample type in each reference included the species and strain of putative probiotic used, its source, the dosage applied (e.g., the colony-forming units (CFU) per unit substrate), the dietary substrate, the type of manipulation (e.g., substrate inoculation, ex situ fermentation), and the impact of the manipulation (e.g., effects on larval survival or mass). For the studies on the impact of BSFL bioconversion on deleterious microbes or microbial toxins, the data charted for each experiment in each reference included the microbe under investigation, the effect of BSFL bioconversion on the levels of the microbe or toxin, the substrate, the duration of the study, whether the study was in vitro or in vivo/in situ, the material sampled (substrate or larvae) in in vivo studies, the method of microbe detection (e.g., qPCR, plating), and the identity of any BSFL gut microbes identified as responsible for the effects on the pathogen. Information organized on the data-charting forms was used to collate and report the state of knowledge on BSFL–microbe interactions and identify the most commonly appearing microbes, methodologies, and results. 

## 3. Results

### 3.1. Review Statistics

**Figure 1:**
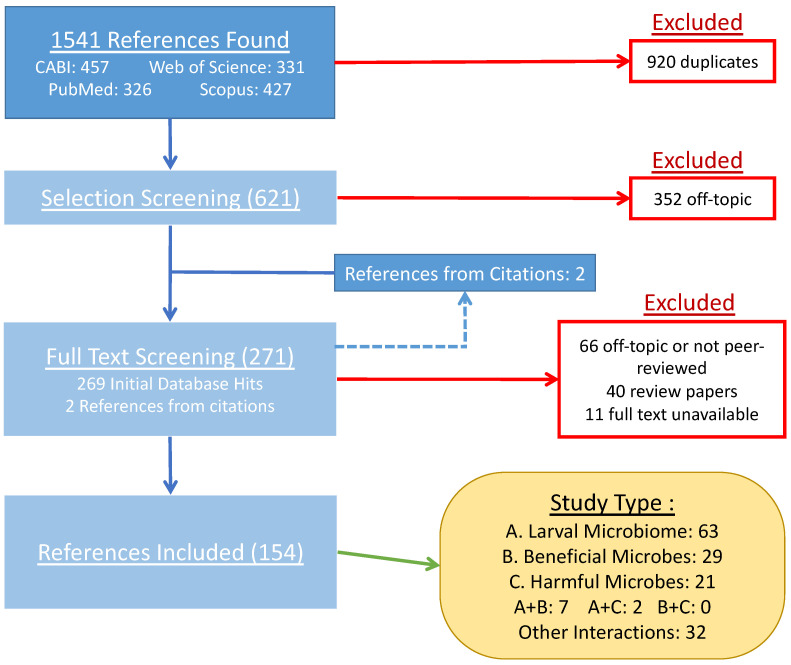
PRISMA flowchart for this study. The studies on BSF–microbe interactions were identified as observational research on the BSFL microbiome, observational or experimental studies looking for microbes that benefit BSFL production when incorporated into the bioconversion system, research on the impact of BSFL bioconversion on pathogenic microbes in the substrate, some combination of the above, or none of the above.

The results of the review are summarized in a PRISMA flowchart (Figure 1). In total, 1541 studies were imported for screening, of which 920 were duplicates. From the rest, 352 were deemed irrelevant [i.e., did not pertain to BSF and microbes] from a title and abstract search, 106 were excluded following a full-text analysis, and full texts could not be obtained for another 11. The following are the results based on the analysis of 154 publications on BSF–microbe interactions. The oldest was the iconic Erickson et al. paper of 2004 which found that BSFL can reduce enteric bacteria levels in chicken manure [20]. Thus, this work spans 20 years of research on black soldier fly interactions with microbes.

### 3.2. Black Soldier Fly Larval Microbiome Diversity

Some insects like termites with nutritionally imbalanced diets are highly dependent on microbial symbionts with digestive functions. Numerous studies have screened the BSFL gut to find such symbionts or core microbes that contribute to their bio-waste digestion ability (Supplementary Table S1). The results clearly show that BSFL gut microbes play important roles in larval digestion. Microbe-free BSFL had reduced substrate reduction and digestion abilities that could be rescued with re-inoculation of BSFL gut homogenates [42]. Whether a conserved, core microbiome is responsible for these effects is less clear. A few genera were suggested to be core microbes within their study populations, where they universally existed in all treatments, but these results were not conserved across multiple studies. Since diet strongly influences gut microbiome structure [43,44,45], this text presents the results of studies using the same or similar diets (intra-diet analysis) to control for diet and minimize its impact in the analysis as much as possible (Table 1). The full data with all the diets are available in Supplementary Table S1 if any reader wishes to perform an inter-diet study. Three categories of diets appeared most frequently and had enough studies for meaningful comparison of their results: the Gainesville diet [46], chicken feed, and manure. For this analysis, possible core microbes are defined as genera with relative abundance above 5%.

Nine studies fed larvae with the Gainesville diet or an approximate formula. Studies with different environmental conditions were included in this analysis, but studies that involved the addition of substances such as extra fiber or flavonoids were excluded to minimize variation between diets as much as possible. Seven of nine screened the gut microbiome with culturing-independent 16S sequencing for bacteria and archaea [43,47,48,49,50,51,52], one used 18S sequencing [53], and one incorporated both 16S and ITS sequencing to also catch eukaryote microbes like fungi [54]. The most commonly occurring microbes were *Dysgonomonas* spp. and *Providencia* spp., appearing in five out of nine studies, followed by *Morganella* spp., *Acinetobacter* spp., *Stenotrophomonas* spp., *Pseudomonas* spp., *Enterococcus* spp., and *Klebsiella* spp., each appearing in three studies. 

Fourteen studies fed their larvae with commercial chicken feed without additional fiber or other substances. Chicken feeds can vary in their ingredients, storage, manufacturer, and purpose (e.g., starter mash, layer hen feed), but nonetheless should be closer to each other in composition and nutrition than they are to substrates like manure or food waste. One study only identified culturable microbes [55], ten applied 16S sequencing [56,57,58,59,60,61,62,63,64,65], one used both 16S sequencing and ITS sequencing [66], one used both 26S sequencing and ITS sequencing [67], and one applied metatranscriptomic sequencing [68]. The most commonly occurring microbes in BSFL fed chicken feed were *Morganella* spp. and *Enterococcus* spp., both appearing in seven out of fourteen studies; followed by *Providencia* spp., which occurred in five studies. The yeast *Trichosporon* sp. occurred in both of the studies that used ITS sequencing.

Fifteen studies used manure as the substrate in 22 treatments (for example, if one study compared manure from various animals or a mixture of two, each would be a separate treatment). Manures vary greatly depending on their source animal and its diet. Still, manures can be grouped together as they have many meaningful similarities: an abundant microbiome, relative depletion of nutrients due to having passed through an animal digestive system, and hygienic concerns. Twelve studies used 16S sequencing [3,22,47,62,63,69,70,71,72,73,74,75], two incorporated both 16S sequencing and ITS sequencing [76,77], and one screened with metatranscriptomic sequencing [68]. The most commonly occurring microbes were *Dysgonomonas* spp., appearing in 10 out of 22 treatments, followed by *Morganella* spp. (9/22 treatments), *Enterococcus* spp. (8/22 treatments), and *Providencia* spp. (6/22 treatments).

Across all these studies, some genera occurred more commonly than others: *Dysgonomonas, Enterococcus, Klebsiella, Morganella,* and *Providencia* (Table 1). This was true even in the one study limited to culturable microbes [55]. However, even among larvae with the same diet type, none of the microbes was found with universal relative abundance higher than 5%. No single genus hit this standard in every study, suggesting that BSFL do not have a globally conserved core microbiome independent of diet. If looking at all BSFL microbiome studies (Supplementary Table S1), the same five genera are the most common and the lack of a conserved core is even more pronounced. To summarize, BSFL are unlikely to obligately depend on specific gut symbionts, but rather BSFL can benefit from interactions with many different microbes. Note too that the most functionally important microbes will not necessarily be the most abundant. For example, *Rhodopseudomonas palustris* induced better growth performance even when its relative abundance was below 5% [78], which demonstrates the limitation of using percentage abundance to identify symbionts. Finally, note that <20% of the reviewed microbiome papers (13 out of 71) used methods that could identify non-bacterial microbes, like ITS metabarcoding or metatranscriptomics, so data on archaea or eukaryotes in BSFL guts are lacking.

Besides diet, other factors can change the BSFL gut microbiome’s structure. Antibiotics in the substrate like chlortetraclycine, gentamicin, nifuroxazide, oxytetracycline, sulfamethoxazole, tetracycline, and tylosin altered the larval gut microbiome composition [79,80,81]. Other substances like sodium chloride, flavonoids, polyethylene, polystyrene, and fibers (including keratin, hemicellulose, cellulose, pectin, and lignin) also changed the microbiome composition when incorporated into the diet [48,82,83,84], as did pre-treatments of the substrate like with alkaline peroxide [85].

Besides diet, variations in the geographical distribution of the collected larvae, larval growth phase, period of starvation, gut region samples, substrate particle size, and moisture content of the substrate also led to dissimilar microbiome structures [22,43,53,86,87,88,89]. The larval gut microbial community can be influenced by biotic factors, such as microbe inoculation [78] or transmission from the previous generation [53]. Pre-fermentation of the diet using larval frass extract from the previous rearing cycle significantly changed the larval gut’s bacterial community and increased the lignocellulosic matter degradation rates in the next cycle by up to 99.95% [90]. Ma et al. established two strains of BSFL adapted to low temperatures, and found that they and the control strain all had different microbiomes when reared under low temperature [49]. An interesting finding from Gorrens et al. implied that a set of larval gut microorganisms reoccurs at specific time points in each rearing cycle within individual facilities using the same rearing parameters [58]. While the microbiome changes over the life of BSFL, these changes and the microbial communities at different points did not vary much when all rearing conditions were kept the same.

### 3.3. Microbes for Improving Black Soldier Fly Larval Performance

Recent reports suggest that specific microbes could further improve the performance of BSFL waste conversion and the quality of the harvested larvae biomass. Certain microbes in the substrate, including some lignocellulose-digesting microbes, will be amplified in the gut, and then their abundance in the substrate increases through the larval frass [91]. Modifying the substrate or larval gut microbiome could thus optimize the BSFL rearing process. This section and Supplementary Table S2 summarize data from studies examining the effects of microbial probiotics, delivered either by substrate inoculation at the same time as the larvae were added or by pre-fermentation of the substrate before larval addition. Bacteria and fungi were both used as probiotics. Some of these probiotics were sourced from natural BSFL colonies and then tested in experiments, while others had exogenous (non-BSF) sources. While some studies tested specific microbe species, others tested inocula with undetermined microbes [23,92,93,94,95] or did not disclose the microbe composition to protect commercial interests [96].

Inoculating the substrate with probiotic microbes often improved the number of larvae surviving to pupation and their mass at harvest [54,97,98,99,100], both of which increase the harvested biomass. Other microbes affected waste conversion and reduction rates. A higher conversion rate signifies more harvested insect biomass under the same substrate consumption rate, meaning the insects could better utilize the nutrients in the substrate. A higher substrate reduction rate implies the insects consumed more substrate and left fewer nutrients behind. Evidence showed that both conversion rate and substrate reduction rate could be improved by inoculating the substrate with probiotics [26,101,102,103,104,105]. In addition to biomass quantity, the value of a BSFL facility’s harvest depends on biomass quality, i.e., nutritional composition. Qualitative properties, fatty acid profiles, amino acid profiles, and larval antimicrobial peptide concentrations have all been modified by introducing microbes into the rearing substrate [78,106]. Chen et al. isolated unidentified, nitrifying bacteria from the BSFL that, when added to a BSFL bioreactor processing swine wastewater, improved larval weight, biomass, and protein gain [92]. Inoculation of probiotics facilitated lipid bioconversion, protein metabolism, vitamin B6 metabolism, purine metabolism, and aflatoxin B1 degradation [25,107,108]. Only a few cases found negative or no effects of microbe inoculation [102,109,110,111], which suggests a positive-result publication bias. 

Pre-fermentation of the substrate had multiple possible benefits, including improved larval weight, nutritional profile, and growth rate; greater substrate reduction and bioconversion efficiency; and reduced greenhouse gas and ammonia emissions [103,110,111,112,113,114,115,116,117,118]. Some of these effects were due to alterations in the substrate’s protein and lipid profiles [119]. BSF adults were also affected by substrate pre-fermentation, showing increased fertility [21]. Positive bias exists here as well, though less severe: Heussler et al. reported insignificant bioconversion improvement after pre-fermentation with yogurt, and reduced adult male size [120]. Note that the highest larval performance was not necessarily with the highest concentration of bacteria [103]. Three studies tested the effect of pre-fermentation for different durations and found that longer pre-fermentation led to reduced BSFL performance and bioconversion rates. One explanation was longer pre-fermentation increasing microbial respiration, reducing the total solids content, and depleting nutrients larvae need to develop [95,110,121].

Thus, while it may take more than a single strain of microbe, it is possible to quantitatively and qualitatively improve BSFL harvest with probiotics. One of the approaches to improve the conversion rate is through enabling digestion of materials like cellulose, hemicellulose, and lignin that the fly larvae themselves cannot digest [54,74,104]. Desirable microbes can be selected from the BSFL gut using qualitative culturomics, as was conducted to isolate cellulolytic strains of *Bacillus altitudinis* and *Klebsiella oxytoca* [122], which can be further studied for probiotic or possibly industrial use [123].

### 3.4. Pathogens, Microbial Toxins, and Antibiotics

Having discussed beneficial microbes, this section looks at the effects of BSFL on pathogenic microbes, microbial toxins, and antibiotics (Supplementary Table S3). The bio-waste fed to BSFL is easily infested by vertebrate pathogens, as well as the [relatively few] known entomopathogens of BSFL themselves [124], like *Beauveria bassiana* [125,126] and the soft-rot pathogen *Paenibacillus thiaminolyticus* [127]. Other potentially harmful substances in substrates include antibiotics, mycotoxins, and heavy metals. Since a major application of the harvested larvae is use as a feed ingredient, the industry must have sufficient knowledge of the potential health risks of BSFL products and how to manage them.

As mentioned before, BSFL conversion usually suppresses pathogen growth or accelerates pathogen reduction [20,59,75,128,129,130,131,132,133], although rebounds can occur [20,134]. Two immune genes in BSFL, *Duox* and *TLR3*, can facilitate pathogen suppression by regulating larval intestinal bacterial community homeostasis [135]. However, contrasting evidence exists where larvae did not reduce the pathogen population, or instead allowed pathogens to multiply [19,136,137,138,139]. The larvae themselves could be contaminated by pathogens and may cause cross-contamination between substrates [20,140]. These conflicting reports demonstrate the necessity of individualized research examining each rearing process or post-harvest sterilization case separately. One study suggested that, in their case, the primary risk of microbial contamination occurred during handling, distributing, and storing the harvested larval meal, and not during BSFL rearing itself [141].

The phenomenon that pathogen suppression continues even after the BSFL are removed from the substrate implies that the larvae may not just destroy pathogens by digesting them, but instead secrete some unknown suppressing substances [19]. Some studies speculated that BSFL-associated microbes are responsible for this pathogen reduction activity. *Trichosporon* spp., *Serratia marcescens, Bacillus subtilis*, and *B. amyloliquefaciens* possibly contributed to the reduction in *Staphylococcus aureus* and *Salmonella* spp. in the substrate in some cases [59,75,129], and *Aspergillus oryzae* and *Lactobacillus plantarum* were suspected to degrade the *Fusarium* mycotoxin in another [115]. Several microbes and a bacteriophage isolated from BSFL demonstrated in vitro antimicrobial activities [65,142,143,144]. Increased abundance of these microbes or their antimicrobial compounds in BSFL-treated substrate could explain post-treatment pathogen suppression. If the gut microbial community of BSFL collectively possesses a broad microbial suppression effect against other microbe species, then this be the source of BSFL’s notable pathogen suppression ability [59,144]. This ability has applications outside bioconversion. For example, whole BSF in the diet have reported antibiotic effects on swine, potentially due to the co-ingested microbiome [9]. Several *Lactobacillus* species isolated from BSFL demonstrated microbial antagonism, pH and bile salt tolerance, and adherence to the poultry intestine, making them good candidates for use as poultry probiotics [10]. 

Many antibiotics are significantly degraded during BSFL waste conversion [79,145,146,147,148], though not always [149,150,151]. Several BSFL gut microbes possess antibiotic degradation ability and antibiotic resistance genes, and are likely at least partly responsible for the observed effects of the bioconversion [81,146,148,152,153,154,155,156]. These microbes could be used for antibiotic degradation without the flies [154,155]. Antibiotic degradation was found to be more efficient when these isolates were applied along with BSFL than when isolates were used alone, implying a synergistic effect between the larvae and microbes [154]. Even when measurable degradation occurs, accumulation of antibiotics in the larvae is still possible [146], so the accumulated antibiotic content in BSFL products should be tested for after harvest. The impact of BSFL or BSFL-associated microbes on the transmission of antibiotic resistance genes is unknown.

Similarly, some mycotoxins can be degraded by BSFL, while some cannot [157,158], and some can accumulate in the larvae, albeit at low levels [159]. In two studies, the concentration of deoxynivalenol increased in the residual substrate after BSFL conversion, though this may have been due to the reduced amount of digestible matter left behind [115,158]. A study comparing sterile and non-sterile BSFL found an 88.7% degradation rate of Aflatoxin B1 in non-sterile larvae, although no bioaccumulation was reported in either treatment [25]. Finally, BSFL gut microbes may help process other toxins. Different microbes isolated from BSFL or certain substrate wastes could promote the conversion or transformation of different heavy metals [62,70,93,160], but heavy metals could also affect the microbiome [161]. BSFL microbes were found to degrade microplastics in one study [162], but showed mixed results in another [48].

## 4. Discussion

The literature on BSF–microbe interactions is robust and growing. While the experiments often differed in their goals, research questions, location, or substrates used, some general conclusions could still be reached. As older reviews have noted [28], BSFL depend in part on their microbiome for survival and digestion. Larvae with sterile guts consistently underperformed those with control or gnotobiotic gut microbiomes in several performance metrics [163]. The results of microbiome studies within a single BSFL facility are often consistent, but will differ from similar studies in a different facility using different substrates or rearing protocols. Regardless of diet, the BSFL gut microbiome frequently includes relatively abundant species of facultatively anaerobic bacteria associated with animal digestive tracts, primarily from the genera *Dysgonomonas*, *Providencia*, *Morganella*, *Enterococcus*, and *Klebsiella* (Table 1). Note that a recent paper re-analyzing published BSFL 16S microbiome data identified *Scrofimicrobium* as another member of this commonly recurring group [29]. In the current review, it only appeared in one study from 2022 [58]. Scrofimicrobium is a recently described genus related to Actinomyces [164], so it is possible but not guaranteed that the Actinomyces spp. in previous studies are also this genus.

What remains unknown is whether the microbes from these most common genera are also the most responsible for the desirable effects of BSFL bioconversion (waste digestion, xenobiotic degradation, etc.). Alternatively, they are commensals better suited for conditions in the BSFL gut compared to other waste microbes. Studies looking at the physiochemical and biomolecular mechanisms of gut colonization by these microbes are needed. Possible commercial or industrial applications of these and other BSFL gut microbes are an underexplored field. In addition, data on the fungi in BSFL guts are relatively lacking, as most microbe studies used bacteria-specific methods like 16S metabarcoding. A few added eukaryote barcodes like ITS or used neutral techniques like metatranscriptomics, and two exclusively fungal studies exist [87,165], but these studies are not enough to draw meaningful conclusions on the role of fungi in the BSFL gut. 

Experiments confirm the use of certain microbes, naturally associated with BSFL or not, as probiotics for BSFL (Supplementary Table S2). Probiotics that succeed in bench-top experiments do not always work when scaled up, however [102]. Before applying any potential probiotics commercially, several factors need to be taken into consideration. The concentration of starting inoculum also must be optimized, as performance does not necessarily improve with higher concentrations, but rather the best performance occurs at intermediate ones [103,117]. Other variables such as the bio-waste composition and the cost of microbe culturing will also affect the compatibility and viability of a strain or a formulation of probiotics. Adding microbes to a substrate could affect the relative abundances of other microbes in the BSFL gut, complicating efforts to find causal relationships between microbes and their effects [78,103]. Given their great potential, further study of BSFL probiotics and substrate modifications like pre-fermentation is advised [166]. 

A notable finding is that none of the studies on BSFL probiotics (Supplementary Table S2) involved any of the most common genera of BSFL gut microbes (Table 1), other than a *Stenotrophomonas* study [25]. That should not be the case: *Morganella*, *Providencia*, *Enterococcus*, *Klebsiella*, and *Dysgonomona* are facultatively anaerobic and culturable. A follow-up hypothesis is that these genera can colonize the gut better than the already studied probiotics. A future direction would be to genetically modify them with desirable traits like lignocelullolytic enzyme production. Also unknown is whether probiotics function through external fermentation of the substrate or through activity inside the BSFL gut itself, as few studies verified gut colonization by the probiotic. These are all examples of siloing, where the probiotics studies focused on the quantitative outcomes without addressing the underlying physiological and microbial ecology questions, and vice versa. 

The potential of isolated BSFL gut microbes as useful products themselves, outside of a BSFL bioconversion context, is still being unraveled, but an increasing number of related studies are expected in the future. Xiao et al. found that fungi and bacteria isolated from BSFL frass can improve the composting of waste without BSFL involvement [167], but other possible uses include enzyme production and environmental decontamination. Similarly, the use of non-microbes to improve the substrate or gut microbiome and produce beneficial effects is understudied. Adding rice straw to a substrate significantly altered the BSFL microbiome as measured from frass samples, but with no effect on larval biomass [168,169]. *Artemesia argyi* flavonoids improved BSFL parameters by regulating the microbiome [84], as did the preservative potassium sorbate [120], while biochar had the opposite effect [120] despite being hypothesized to benefit compost microbes [170].

Conclusions about BSFL elimination of pathogenic microbes, including viruses [171,172], or their toxins can be made, but they are not universal. BSFL bioconversion usually reduces pathogen loads, but not always (Supplementary Table S3). Positive-result bias in the literature may further affect these data. Different rearing systems and conditions, substrates, post-harvesting strategies, and even BSFL gut microbiomes all impact whether or not different pathogens will be eliminated from a substrate through bioconversion and/or contaminate final BSFL products to an unsafe level. Thus, as with any food or feed production system, safety testing should be carried out and hygienic procedures followed. Importantly, the post-harvesting sterilization, handling, storage, and transport of any BSFL product may have a more significant impact on pathogen risks than aspects of the rearing and bioconversion itself [173].

Methods of studying the BSFL microbiome are not standardized, and will likely continue to change as technologies change. However, the authors have some methodological suggestions. Future BSFL gut microbiome studies should focus on specific diets, and more should check for eukaryotic microbes. Phylum-level microbiome assays are of limited usefulness [174]. The numerous BSFL microbiome studies sampled larvae at various growth stages or did not determine the instars. However, the gut microbiome changes between growth stages (potentially in a reoccurring and predictable pattern [58], though more research is needed to verify this observation). BSFL microbiome studies should avoid pooling samples from different instars of BSFL together, as this can obscure important differences, and instead analyze different instars separately to cover the full diversity of the microbiome in the larval life cycle. Larval weights are not enough to determine the instar. Head capsule size in combination with length and weight is better [175]. Bonelli et al. further found distinct divisions in the midgut of BSFL [176], indicating that deciding which part of the gastrointestinal tract to sample is also critical. If the gut is dissected or pulled out, care should be taken to prevent loss of part of the foregut or hindgut in the process. Using surface-sterilized whole insects would solve that problem.

## 5. Conclusions

To summarize the results of two decades of research from around the globe on BSFL–microbe interactions, this review reached the following conclusions: (1) The BSFL gut microbe undoubtably plays a role in their performance through the production of digestive enzymes plus antimicrobial and anti-xenobiotic functions. (2) There is no universally conserved core microbiome in BSFL, though the facultatively anaerobic genera *Dysgonomonas, Enterococcus, Klebsiella, Morganella,* and *Providencia* are repeatedly found in relatively high abundance. (3) The exact roles and functions of these common microbes are not always known, nor is there reason to believe these are the most important microbes in the BSFL gut beyond their relative abundance. (4) Data on non-bacterial microbes (archaea, fungi, protozoa) and their interactions with BSFL are lacking, representing a gap in the literature. Several microbes have a beneficial effect on BSFL or their bioconversion rates when added to the substrate with or before the larvae. (5) Little to no overlap exists between the commonly isolated or identified microbes from the gut and the microbes so far tested as probiotics or pre-fermentation microbes, which represents another major gap in the literature that should be filled with future research. (6) Whether probiotics function through colonization of the BSFL gut or solely through exogenous action on the substrate has rarely been examined, and represents a third major knowledge gap. (7) BSFL are not always capable of eliminating pathogens, and so each individual facility should check its system for appropriate risk factors independently of what other studies in other locations have claimed. (8) Knowledge on the basic digestive physiology of the BSFL has not kept pace with the number of next-generation microbiome studies, limiting the ability to design useful experiments and correctly interpret results.

The following research priorities for BSFL–microbe interaction studies are identified: inventories of eukaryotes in the microbiome, higher-resolution studies of how the microbiome differs with gut segment and over larval development, testing of the common microbes for probiotic potential, and identifying the mechanisms behind observed probiotic activities and physiology of probiotics in the BSFL gut.

## Figures and Tables

**Table 1:** Black soldier fly gut bacterial genera and the number of experiments (n) out of the total in which their relative abundance was >5%.

Bacterial Clade		Diet			Features	
Class: Order: Family	Genus	G (9)	C (14)	M (22)	Gram	O_2_ Needs
Actinomycetes: Actinomycetales: Actinomycetaceae	*Actinomyces*	1	3	0	+	Facultative Anaerobe
Bacilli: Lactobacillales: Enterococcaceae	*Enterococcus*	3	7	8	+	
Bacilli: Lactobacillales: Leuconostocaceae	*Weisella*	0	3	0	+	
Bacteroidia: Bacteroidales: Dysgonomonadaceae	*Dysgonomonas*	5	3	10	-	
Clostridia: Clostridiales: Clostridiaceae	*Clostridium*	0	0	5	+	Obligate Anaerobe
Clostridia: Eubacteriales: Peptostreptococcaceae	*Paeniclostridium*	0	0	3	+	
	*Romboutsia*	0	0	5	+	
	*Terrisporobacter*	0	0	4	+	
Gammaproteobacteria: Enterobacterales: Enterobacteriaceae	*Klebsiella*	3	3	1	-	Facultative Anaerobe
Gammaproteobacteria: Enterobacterales: Morganellaceae	*Morganella*	3	7	9	-	
	*Proteus*	0	3	2	-	
	*Providencia*	5	5	6	-	
Gammaproteobacteria: Pseudomonadales: Moraxellaceae	*Acinetobacter*	3	0	0	-	Obligate Aerobe
Gammaproteobacteria: Pseudomonadales: Pseudomonadaceae	*Pseudomonas*	3	2	2	-	
Gammaproteobacteria: Xanthomonadales: Xanthomonadaceae	*Stenotrophomonas*	3	1	0	-	

Table sorted by clade. Fungi are not included as few studies examined eukaryotes while all examined bacteria. Diets are the following: G[Gainesville Diet], C[Chicken Feed], and M[Manure]. For manure, the 22 experiments are described in 15 publications. The complete data are in Supplementary Table S1.

## Data Availability

The original contributions presented in the study are included in the article/Supplementary Material. Further inquiries can be directed to the corresponding author.

## Supplementary Materials

The following supporting information can be downloaded at https://www.mdpi.com/article/10.3390/ani14223183/s1: Table S1: Summary of investigations into BSFL microbiomes; Table S2: Summary of investigations into potential BSFL probiotics; Table S3: Pathogens affected by BSFL conversion of substrate or by isolated BSFL gut microbes in vitro.
